# Leptomeningeal Metastases in a Patient with Castration-Resistant Prostate Cancer

**DOI:** 10.1155/2020/5627548

**Published:** 2020-09-12

**Authors:** Takuya Koie, Yasuhiro Hashimoto, Yuichiro Suzuki, Yoshiomi Hatayama, Futoshi Kimura

**Affiliations:** ^1^Department of Urology, Gifu University Graduate School of Medicine, Gifu, Japan; ^2^Department of Urology, Hirosaki University Graduate School of Medicine, Aomori, Japan; ^3^Department of Radiation Oncology, Hirosaki University Graduate School of Medicine, Aomori, Japan; ^4^Department of Anesthesiology, Hirosaki University Graduate School of Medicine, Aomori, Japan

## Abstract

A 42-year-old man visited a community hospital with chief complaints of lumbago and dyschesia. Computed tomography (CT) showed multiple lung, lymph node, and bone metastases and the irregular enlarged prostate with urinary bladder invasion. Serum prostate-specific antigen (PSA) was 544.0 ng/mL. Histological evaluation showed adenocarcinoma with the Gleason score 5 + 4, and the clinical stage was T4N1M1c as an initial diagnosis. Although androgen deprivation therapy was performed immediately, he had castration-resistant PCa after 3 months. Therefore, he received 6 courses of docetaxel chemotherapy every 3 weeks. Serum PSA was decreased to 0.2 ng/mL, and multiple metastases and prostate size were obviously reduced based on CT. He underwent robot-assisted radical prostatectomy and radiation therapy for prostatic fossa and multiple metastases. Although serum PSA level remained low, CT showed multiple liver metastases after 3 years from surgery. He received the combination therapy of cisplatin and etoposide (PE) every 4 weeks. Liver metastases had complete response. However, he visited our hospital with complaint of vomiting and a right drooping eyelid after 6 weeks from withdrawal of PE therapy. T2-weighted magnetic resonance imaging revealed multiple leptomeningeal metastases (LM). He received RT for the brain and was administered amrubicin. However, he died of PCa after 6 weeks from the diagnosis of LM.

## 1. Introduction

Prostate cancer (PCa) is the most commonly diagnosed neoplasm in male worldwide, and the vast majority of patients with PCa developed bone and/or lymph node metastases [1]. Conversely, leptomeningeal metastasis (LM) from genitourinary cancer is rare, especially from PCa [1, 2]. Herein, we report a patient with castration-resistant PCa (CRPC) who developed LM after multidisciplinary therapy.

## 2. Case Reports

**Figure 1:**
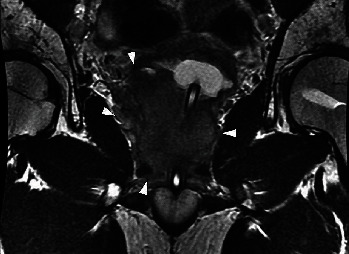
The irregular enlarged prostate with urinary bladder invasion (arrows).

**Figure 2:**
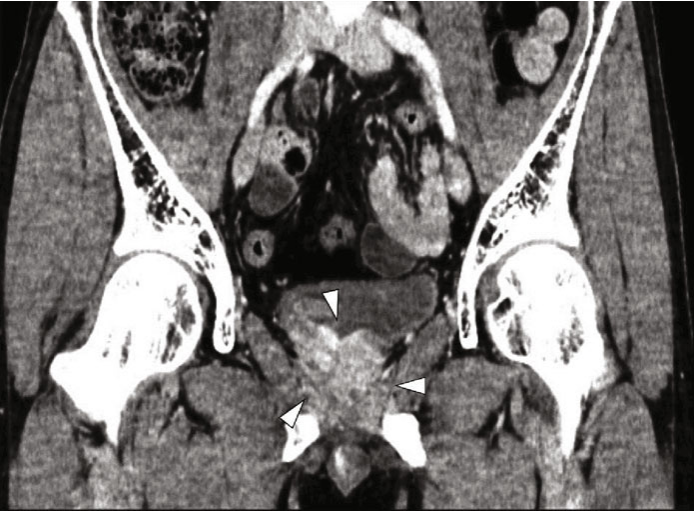
Prostate size was obviously reduced based on computed tomography after the administration of docetaxel (arrows).

**Figure 3:**
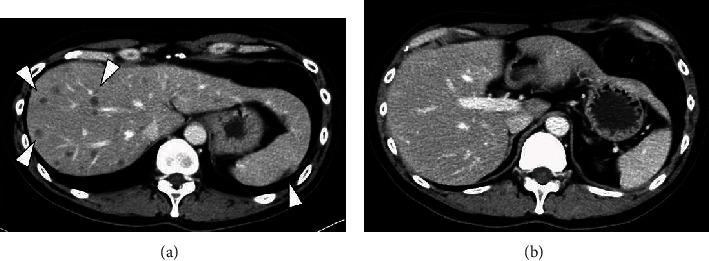
Liver metastases before and after the combination chemotherapy of cisplatin and etoposide: (a) computed tomography showed multiple liver metastases (arrows); (b) liver metastases had achieved complete response in a patient who received combination chemotherapy of cisplatin and etoposide.

**Figure 4:**
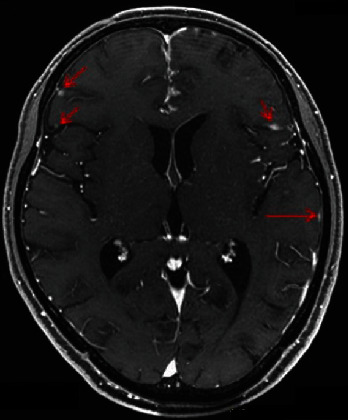
T2-weighted magnetic resonance imaging revealed multiple leptomeningeal metastases at bilateral trigeminal, facial, and abductor nerves and surrounding tissues of the brain stem and cervical spinal cord (arrows).

A 42-year-old man visited a community hospital with chief complaints of lumbago and dyschesia. Computed tomography (CT) showed multiple lung, lymph node, and bone metastases and the irregular enlarged prostate with urinary bladder invasion (Figure 1). Serum prostate-specific antigen (PSA) was 544.0 ng/mL (normal range: <4.0 ng/mL), so that he underwent transrectal prostate biopsy. Histological evaluation showed adenocarcinoma with the Gleason score 5 + 4, and the clinical stage was T4N1M1c as an initial diagnosis. Although androgen deprivation therapy (ADT) was performed immediately, he had CRPC after 3 months. Therefore, he received 6 courses of docetaxel (70 mg/m^2^) chemotherapy every 3 weeks. Serum PSA was decreased to 0.2 ng/mL, and multiple metastases and prostate size were obviously reduced based on CT (Figure 2). He underwent robot-assisted radical prostatectomy after 10 months from ADT. Pathological diagnosis was PCa with a positive surgical margin. One month later, radiation therapy (RT) was performed for prostatic fossa (total 60 Gy), iliac lymph nodes (total 54 Gy), and lung metastases (total 72 Gy). Nadir PSA level was 0.09 ng/mL after surgery and RT. However, serum PSA level gradually increased. Therefore, he received several treatment modalities for PCa, including enzalutamide, abiraterone, cabazitaxel, and rechallenge of docetaxel. Although serum PSA level remained low (0.75 ng/mL), CT showed multiple liver metastases after 3 years from surgery (Figure 3(a)). At the time of liver metastases, several tumor markers showed: PSA 3.01 ng/mL, neuron-specific enolase 8.9 ng/mL (normal range: <16.3 ng/mL), and pro-gastrin-releasing peptide 58.1 pg/mL (normal range: <81 pg/mL). The patient did not undergo biopsy for liver metastases. However, we consider that his cancer cells might increase aggressiveness by stimulating angiogenesis even though tumor markers were normal. Therefore, we selected the regimen of cisplatin-based combination chemotherapy. He received 6 courses of combination therapy of cisplatin (80 mg/m^2^) and etoposide (100 mg/m^2^) (PE) every 4 weeks. Liver metastases had complete response (Figure 3(b)). Therefore, chemotherapy was discontinued temporarily despite continuous ADT. However, he visited our hospital with complaint of vomiting and a right drooping eyelid after 6 weeks from withdrawal of PE therapy. T2-weighted magnetic resonance imaging (MRI) revealed multiple LM at bilateral trigeminal, facial, and abductor nerves and surrounding tissues of the brain stem and cervical spinal cord (Figure 4). He received RT for the brain (total 30 Gy) and was administered amrubicin (30 mg/m^2^). However, he died of PCa after 6 weeks from the diagnosis of LM.

## 3. Discussion

LM results from the dissemination of cancer cells to both the leptomeninges and cerebrospinal fluid compartment [3]. LM is diagnosed in 4-15% of patients with solid tumors [3]. Adenocarcinoma is the most frequent histological finding, and the breast (5%), lung (11%), and skin (melanoma) (20%) are the most common primary sites to LM [2, 3]. Conversely, LM from genitourinary tract cancer is very rare, especially in PCa. The vast majority of PCa patients with metastases will eventually die from bone metastases, while spread to the lungs, liver, and brain is a later and less common event [2]. Clinical reviews report the incidence of central nervous system (CNS) metastasis from PCa to be less than 1%, while on autopsy, it ranges from 1 to 11% [2].

LM is usually a late manifestation of systemic disease and most often occurs in patients after multidisciplinary therapies [2]. Cancer cells spread to the leptomeninges by several reasons: (1) arterial or venous circulation, (2) initiation of contact of leptomeninges with the tumor, and (3) centripetal progression along perineural or perivascular spaces [3]. Once in the subarachnoid space, tumor cells can seed multiple areas of the CSF circulation [3]. In this case, multiple liver metastases were identified before LM. Therefore, the authors consider that PCa cells may disseminate to the leptomeninges through hematogenous spread. In this case, we opted to perform RARP for the prostate even though he had multiple metastases. Faiena et al. reported that patients with metastatic disease who received definitive local therapy had greater survival than patients who received no treatment [4]. In addition, a recent Surveillance, Epidemiology, and End Results database study analyzed the cancer-specific survival (CSS) in patients with metastatic PCa who received definitive local therapy or observation [5]. The 5-year OS and predicted cancer-specific survival were significantly higher in patients who underwent RP (67.4 and 75.8%, respectively) or brachytherapy (52.6 and 61.3%, respectively) compared with patients with no treatment (22.5 and 48.7%, respectively) (*P* < 0.001) [5]. Furthermore, definitive therapy for the prostate was significantly associated with decreased cancer-specific mortality (*P* < 0.01) [5]. Therefore, we performed RARP for the patient in spite of multiple metastases.

The diagnosis of LM is made either by identifying malignant cells in CSF or by gadolinium-enhanced MRI, although there are high rates of false-negative results with both methods [6]. To date, gadolinium-enhanced MRI represents the best imaging modality for the detection of LM [6].

There is no standard treatment for LM [2]. The typical treatment for LM includes RT and systemic chemotherapy. However, chemotherapy is of limited value because of the blood brain barrier. Therefore, prognosis for PCa patients with LM is poor, and these patients do not survive more than a few weeks to months following the diagnosis of LM [6]. In this case, the patient died of PCa after 6 weeks from the diagnosis of LM. Although LM is rare, early neurological symptoms may help the oncologists to make an early diagnosis of LM.
